# Discovery of natural products capable of inducing porcine host defense peptide gene expression using cell-based high throughput screening

**DOI:** 10.1186/s40104-020-00536-0

**Published:** 2021-01-11

**Authors:** Jing Wang, Wentao Lyu, Wei Zhang, Yonghong Chen, Fang Luo, Yamin Wang, Haifeng Ji, Guolong Zhang

**Affiliations:** 1 Institute of Animal Husbandry and Veterinary Medicine, Beijing Academy of Agriculture and Forestry Sciences, Beijing, China; 2 Sino-US Joint Laboratory of Animal Science, Beijing Academy of Agriculture and Forestry Sciences, Beijing, China; 3 Institute of Quality and Standard for Agro-products, Zhejiang Academy of Agricultural Sciences, Hangzhou, China; 4 College of Agriculture, Ningxia University, Yinchuan, China; 5 Department of Animal and Food Sciences, Oklahoma State University, Stillwater, OK USA

**Keywords:** Antibiotic alternatives, High throughput screening, HDP inducers, Host defense peptides, Natural products

## Abstract

**Background:**

In-feed antibiotics are being phased out in livestock production worldwide. Alternatives to antibiotics are urgently needed to maintain animal health and production performance. Host defense peptides (HDPs) are known for their broad-spectrum antimicrobial and immunomodulatory capabilities. Enhancing the synthesis of endogenous HDPs represents a promising antibiotic alternative strategy to disease control and prevention.

**Methods:**

To identify natural products with an ability to stimulate the synthesis of endogenous HDPs, we performed a high-throughput screening of 1261 natural products using a newly-established stable luciferase reporter cell line known as IPEC-J2/*pBD3*-luc. The ability of the hit compounds to induce HDP genes in porcine IPEC-J2 intestinal epithelial cells, 3D4/31 macrophages, and jejunal explants were verified using RT-qPCR. Augmentation of the antibacterial activity of porcine 3D4/31 macrophages against a Gram-negative bacterium (enterotoxigenic *E. coli*) and a Gram-positive bacterium (*Staphylococcus aureus*) were further confirmed with four selected HDP-inducing compounds.

**Results:**

A total of 48 natural products with a minimum Z-score of 2.0 were identified after high-throughput screening, with 21 compounds giving at least 2-fold increase in luciferase activity in a follow-up dose-response experiment. Xanthohumol and deoxyshikonin were further found to be the most potent in inducing *pBD3* mRNA expression, showing a minimum 10-fold increase in IPEC-J2, 3D4/31 cells, and jejunal explants. Other compounds such as isorhapontigenin and calycosin also enhanced *pBD3* mRNA expression by at least 10-fold in both IPEC-J2 cells and jejunal explants, but not 3D4/31 cells. In addition to *pBD3*, other porcine HDP genes such as *pBD2, PG1-5*, and *pEP2C* were induced to different magnitudes by xanthohumol, deoxyshikonin, isorhapontigenin, and calycosin, although clear gene- and cell type-specific patterns of regulation were observed. Desirably, these four compounds had a minimum effect on the expression of several representative inflammatory cytokine genes. Furthermore, when used at HDP-inducing concentrations, these compounds showed no obvious direct antibacterial activity, but significantly augmented the antibacterial activity of 3D4/31 macrophages (*P <* 0.05) against both Gram-negative and Gram-positive bacteria.

**Conclusions:**

Our results indicate that these newly-identified natural HDP-inducing compounds have the potential to be developed as novel alternatives to antibiotics for prophylactic and therapeutic treatment of infectious diseases in livestock production.

## Introduction

Weaning piglets encounter multiple stressors that render them more vulnerable to gastrointestinal infections, manifesting mainly as diarrhea, which severely endangers their health and growth performance [1]. Antibiotics have been used routinely for growth promotion and infection prevention in the swine industry. However, such a practice has been linked to the development of antimicrobial resistance in humans. Because of public health and safety concerns, antibiotics are being gradually phased out in livestock production worldwide. Novel, alternative antimicrobial strategies are urgently needed to ensure the health of livestock animals including piglets.

Host defense peptides (HDPs) are antimicrobial peptides that are produced mainly by various epithelial and phagocytic cells [2, 3]. They are important components of the innate immune system and play essential roles in pathogen elimination. HDPs have been widely studied for their broad-spectrum antimicrobial and immunomodulatory activities. Synthetic HDPs have been developed as feed additives to promote growth performance, nutrient digestion, and intestinal health [4, 5]. However, sensitivity to protease digestion and high production costs have limited their application [6]. Enhancing endogenous HDP synthesis has been confirmed to contribute to the treatment of diseases and infections caused by bacteria such as *Shigella* and enteropathogenic *E. coli* [7–9], with potential to enhance the intestinal homeostatic balance in livestock animals [10]. A number of synthetic and natural small-molecule compounds have been identified to be capable of inducing HDP gene expression [11–14].

Natural products are preferred feed additives because many of them have anti-inflammatory, anti-oxidative, and antibacterial activities, showing promise in disease control and prevention [15]. Several classes of natural products such as short-chain fatty acids, vitamin D, nicotinamide, sugars, branched-chain amino acids, bile acids, zinc, and phytochemicals such as forskolin, sulforaphane, curcumin, resveratrol, pterostilbene, and polydatin have been identified as HDP inducers [11–14]. Although maintaining histone hyper-acetylation status of HDP gene promoters is largely responsible for the HDP-inducing activity of a few natural products, multiple other mechanisms also exist [10–14], which is expected, given structural and functional diversities of such a large group of compounds.

To facilitate the identification of HDP-inducing compounds, we and other have developed cell-based high-throughput screening (HTS) assays for specific applications in humans, poultry, and pigs [16–18]. We have screened a library of 584 natural products and identified a number of HDP-inducing compounds [17, 18]. Using our newly-established IPEC-J2/*pBD3-*luc cell line, which is the porcine IPEC-J2 intestinal epithelial cell line with stable integration of a luciferase reporter plasmid under the control of porcine *pBD3* gene promoter [18], we screened in the present study a library of 1261 natural products, which was custom-made and non-redundant from the 584 compounds that were screened earlier [17, 18]. The objective of this study was to discover additional and perhaps more potent, natural HDP inducers for livestock use. As a result, we identified a number of hit compounds, which were further validated for their ability to induce multiple HDP genes in different porcine cell lines and primary intestinal explants. Augmentation of the antibacterial activity of porcine cells by several selected compounds were also confirmed. Therefore, these HDP-inducing natural products have potential for further development as novel agents for control and prevention of infectious diseases.

## Materials and methods

### Cell lines and culture conditions

A porcine intestinal epithelial cell line, IPEC-J2, was cultured in DMEM/F12, a 1:1 mixture of Dulbecco’s modified Eagle’s medium and Ham’s F-12 (Gibco™, Thermo Fisher Scientific, Waltham, MA, USA) supplemented with 10% fetal bovine serum (FBS; Gibco™), streptomycin (100 μg/mL), penicillin (100 U/mL), and 1% ITS premix (5 μg/mL insulin, 5 μg/mL transferrin, 5 ng/mL selenium) (ScienCell, San Diego, CA, USA) at 37 °C in an atmosphere of 5% CO_2_ and 95% air and 90% humidity. The stable luciferase reporter cell line, IPEC-J2/*pBD3-*luc, was developed as we described [15] and maintained in the same medium as IPEC-J2 with additional supplemtion of 1 μg/mL puromycin. A porcine lung alveolar macrophage cell line, 3D4/31 (ATCC CRL-2844), was cultured in Roswell Park Memorial Institute (RPMI 1640, Thermo Fisher Scientific) supplemented with 10% FBS, streptomycin (100 μg/mL), penicillin (100 U/mL), and sodium pyruvate (1 mmol/L) at 37 °C in an atmosphere of 5% CO_2_ and 95% air and 90% humidity. Cells were subcultured in complete medium every 3–4 day.

### Chemicals and the natural product library

A collection of 1261 pure, unique, natural compounds isolated from plants, animals, and microorganisms with known biological activities for use in drug discovery, pharmacological studies, and stem cell differentiation was purchased from Target Molecule (Shanghai, China). The library includes more than 30 different types of chemicals including, but not limited to, alkaloids, limonoids, sesquiterpenes, diterpenes, pentacyclic triterpenes, and sterols. The compounds were provided as 10-mmol/L stocks in DMSO.

### HTS assay

A HTS assay was conducted as we previously described [15]. Briefly, IPEC-J2/*pBD3-*luc cells were seeded in 96-well plates at 2 × 10^4^ cells/well. After overnight culture, the cells were stimulated with individual compounds at a final concentration of 20 μmol/L for 24 h. Luciferase activity was measured using the Steady-Glo Luciferase Assay System (Promega, Madison, WI, USA), in a L-Max II Luminescence Microplate Reader (Molecular Devices, Sunnyvale, CA, USA). To assess cell viability, alamarBlue Reagent (Thermo Fisher Scientific) was added to each well 4 h prior to the luciferase assay to the final concentration of 0.2%. Fluorescence was quantified in an FLx80 Microplate Fluorescence Reader (BioTek Instruments, Winooski, VT, USA), using an excitation wavelength of 545 nm and an emission wavelength of 590 nm. For each compound, relative luciferase activity was normalized to cell viability. For the selection of active compounds, we calculated the Z-score as follows: $\mathrm{Z}=\frac{x-\upmu}{\sigma },$ where *x* is the relative luciferase activity for an individual compound, *μ* is the mean luciferase activity for all compounds tested, and *σ* is the standard deviation for all compounds tested as described [19]. A compound with a Z-score of ≥2.0, which means that luciferase activity is two standard deviations above the mean, was considered a hit.

### Secondary screening and validation of the hit compounds

Compounds with a normalized Z-score of ≥2.0 were further assayed at three different concentrations (5, 20, and 80 μmol/L) using stable IPEC-J2/*pBD3-*luc cells in 96-well plates, as described above. After normalization to cell viability, the fold change in luciferase activity relative to that in non-stimulated control cells was calculated for each compound. Compounds that showed at least 2-fold increase at any of the three concentrations were further assayed for their HDP-inducing activity in parental porcine IPEC-J2 intestinal epithelial cells (1.25 × 10^5^ cells/well) and porcine 3D4/31 alveolar macrophage cells (4 × 10^5^ cells/well) in 12-well tissue culture plates (Costar, Corning, Corning, NY, USA). After overnight growth, they were exposed to each compound at three concentrations (5, 20, and 80 μmol/L) in duplicate for 24 h, followed by total RNA isolation and RT-qPCR as described below. Non-treated cells were served as a control. The assays were performed at least three times.

### Total RNA isolation and RT-qPCR

After stimulation, cells were lysed in RNAzol RT (Molecular Research Center, Cincinnati, OH), followed by total RNA extraction, according to the manufacturer’s instructions. RNA concentration was assessed using a NanoDrop spectrophotometer (Thermo Fisher Scientific), while RNA quality was confirmed by the A_260_/A_280_ and A_260_/A_230_ ratios. An iScript™ cDNA Synthesis Kit (Bio-Rad, Hercules, CA) was used for cDNA synthesis according to the manufacturer’s instructions. The qPCR was performed using iTaq™ Universal SYBR® Green Supermix (Bio-Rad) in a QuantStudio 3 Real-Time PCR System (Thermo Fisher Scientific). The qPCR program was as follows: 95 °C for 10 min followed by 40 cycles of 95 °C for 30 s, 60 °C for 30 s, and 72 °C for 20 s. Porcine gene primers were designed and the sequences were listed in Table 1. The expression levels of various porcine HDP genes were normalized to that of glyceraldehyde-3-phosphate dehydrogenase (*GAPDH*), whose expression level was not altered by any of the compounds applied. The relative fold changes in gene expression were calculated using the 2^–∆∆Ct^ method [20].

**Table 1:** Primers used in this study

Gene	Forward primer (5'→3')	Product size, bp	Accession number
*GAPDH*	Forward: GCTACACTGAGGACCAGGTTG Reverse: CCTGTTGCTGTAGCCAAATTC	146	XM_021091114.1
*pBD2*	Forward: TGTCTGCCTCCTCTCTTCC Reverse: AACAGGTCCCTTCAATCCTG	149	NM_214442.2
*pBD3*	Forward: CCTTCTCTTTGCCTTGCTCTT Reverse: GCCACTCACAGAACAGCTACC	163	XM_021074698.1
*pEP2C*	Forward: ACTGCTTGTTCTCCAGAGCC Reverse: TGGCACAGATGACAAAGCCT	92	XM_003362076.4
*PG1–5*	Forward: ACGGTGAAGGAGACTGTG Reverse: CGCAGAACCTACGCCTACAA	196	XM_021070622.1
*IL1β*	Forward: GCCCTGTACCCCAACTGGTA Reverse: CCAGGAAGACGGGCTTTTG	61	NM_001302388.2
*IL8*	Forward: TTCGATGCCAGTGCATAAATA Reverse: CTGTACAACCTTCTGCACCCA	176	NM_213867.1
*TNFα*	Forward: CCCCTCTGAAAAAGACACCA Reverse: TCGAAGTGCAGTAGGCAGAA	180	NM_214022.1

### Dose- and time-dependent induction of multiple porcine HDP genes

To determine the optimal concentration and duration of a natural product for HDP induction, IPEC-J2 cells were exposed to xanthohumol, isorhapontigenin, or calycosin at 5, 10, 20, 40, 80, 160, or 320 μmol/L and deoxyshikonin at 0.3125, 0.625, 1.25, 2.5, 5, 10, or 20 μmol/L in duplicate for 24 h, followed up total RNA isolation and RT-qPCR. Once the optimal concentration was determined, IPEC-J2 cells were exposed to each compound at the optimal concentration in duplicate for 6, 12, 24, and 48 h prior to RT-qPCR analysis. Non-treated cells were served as a control. The experiments were performed at least three times.

### *Ex vivo* confirmation of HDP induction using porcine intestinal explants

To verify the ability of individual compounds to induce HDP expression in primary porcine intestinal cells, jejunal segments were obtained from 5-week-old Big White × Large White crossbred piglets (7.85 ± 0.23 kg) that were weaned at 28 d. To prepare jejunal explants, external *tunica muscularis* was removed from the mid-jejunum, and segments of approximately 0.5 cm × 0.5 cm were excised with surgical scissors and washed thoroughly in cold PBS. The explants were then placed in individual wells of 6-well plates containing 4 mL of RPMI 1640 medium supplemented with 10% FBS, 20 mmol/L HEPES, 100 μg/mL gentamicin, 100 U/mL penicillin, and 100 μg/mL streptomycin. The explants were treated in triplicate with different compounds at optimal concentrations at 37 °C for 24 h under an atmosphere of 5% CO_2_ and 95% air and 90% humidity. After stimulation, total RNA was isolated and used for RT-qPCR analysis of porcine HDP gene expression as described above.

### Detection of proinflammatory cytokine expression by natural products

To evaluate the influence of natural products on proinflammatory cytokine gene expression, IPEC-J2 cells were treated in triplicate with 40 μmol/L xanthohumol, 80 μmol/L isorhapontigenin, 5 μmol/L deoxyshikonin, or 80 μmo/L calycosin for 3 h, 6 h, and 24 h, followed by analysis of the mRNA levels of *IL1β*, *IL8*, and *TNFα* using RT-qPCR.

### Augmentation of the antimicrobial activity of porcine 3D4/31 alveolar macrophages

The antibacterial activities of porcine 3D4/31 cells treated with HDP-inducing compounds were assessed as we previously described [15, 16, 21], with slight modifications. Porcine 3D4/31 cells were cultured in 6-well plates and then stimulated in duplicate with 40 μmol/L xanthohumol, 80 μmol/L isorhapontigenin, 5 μmol/L deoxyshikonin, or 80 μmol/L calycosin for 24 h. Then, the cells were collected, washed twice with calcium- and magnesium-free Hank’s balanced salt solution, and resuspended in 100 μL of water. The cells were frozen at − 80 °C for 20 min, thawed on ice, and sonicated for 30 s. The lysates were centrifuged at 12,000×*g* at 4 °C for 10 min. The supernatant (50 μL) was incubated with another 50 μL of F4^+^ enterotoxigenic *E. coli* (F4^+^ ETEC, CVCC225) or *Staphylococcus aureus* (CVCC546) at 2.5 × 10^5^ CFU/mL in 20% trypticase soy broth containing 1 mmol/L NaH_2_PO_4_ and 25 mmol/L NaHCO_3_ in a 96-well plate, at 37 °C. The bacterial turbidity at 600 nm was measured at 3, 6, 12, and 24 h using a Multiskan FC instrument (Thermo Fisher Scientific).

### Minimum inhibitory concentration (MIC) assay

The MICs of xanthohumol, isorhapontigenin, deoxyshikonin, and calycosin were determined using a standard broth microdilution assay as recommended by National Committee for Clinical Laboratory Standards (NCCLS). ETEC and *S. aureus* were streaked onto trypticase soy (Thermo Fisher Scientific) agar plates. One or two individual colonies were cultured to the mid-log phase in trypticase soy broth under shaking at 220 r/min at 37 °C. The bacterial cells were then diluted to 5 × 10^5^ CFU/mL in Mueller Hinton Broth (Thermo Fisher Scientific). After dispensing 75 μL/well in a 96-well tissue-culture plate, 25 μL of each compound was added to final concentrations of 0.625, 1.25, 2.5, 5, 10, 20, 40, 80, 160, and 320 μmol/L in triplicate. After overnight incubation at 37 °C, the lowest concentration of the compound that resulted in no visible bacterial growth was considered the MIC.

### Statistical analysis

Data were presented as the means ± standard errors of the mean (SEM) and were processed using GraphPad Prism version 6 (GraphPad Software, San Diego, CA, USA). Means were compared using unpaired Student’s two-tailed *t*-test. *P* < 0.05 was considered significant.

## Results

### Identification of natural HDP-inducing compounds

**Fig. 1:**
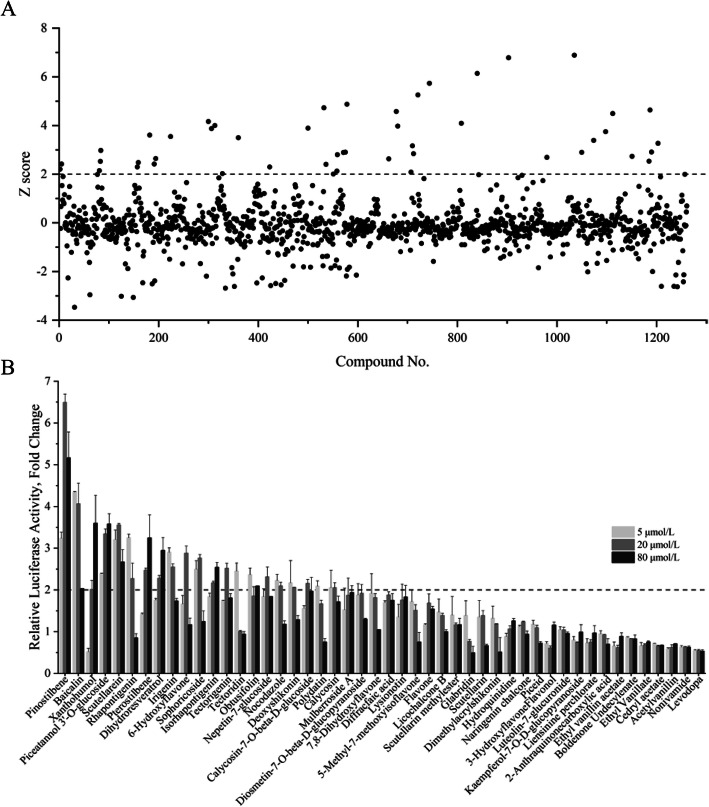
High throughput screening of natural compounds to induce *pBD3* gene expression. **a**, Z-scores of 1261 natural compounds following a primary screening. Stable IPEC-J2/*pBD3-*luc luciferase reporter cells were stimulated with natural compounds at 20 μmol/L in 96-well plates for 24 h. Four hours prior to the luciferase assay, cell viability was measured using alamarBlue cell viability assay reagent. The luciferase activity was measured using a Steady-Glo Luciferase Assay System and was normalized to cell viability before the Z-score was calculated. **b**, Secondary screening based on luciferase activity. IPEC-J2/*pBD3-*luc cells were stimulated with natural compounds at 5, 20, or 80 μmol/L in 96-well plates for 24 h. The fold change in the luciferase activity induced by each compound relative to that in non-stimulated control cells was calculated

A total of 1241 natural small-molecule compounds were screened for their ability to induce porcine *pBD3* expression using IPEC-J2/*pBD3-*luc cells. As a result, 48 compounds were identified with a minimum Z-score of 2.0 (Fig. 1a). Notably, more than 20 compounds had a Z-score of less than − 2.0, suggestive of their potential suppressive effect on *pBD3* expression.

### Secondary screening of the hit compounds

Dose-response experiments were further conducted in stable IPEC-J2/*pBD3-*luc reporter cells to validate the *pBD3*-inducing activity of the 48 newly-identified hits. Most compounds indeed increased luciferase activity, with 21 compounds showing a > 2-fold increase for at least one of the three concentrations examined (5, 20, and 80 μmol/L) (Fig. 1b). These 21 compounds were mainly comprised of flavonoids and phenols (Table 2) and further selected for confirming their HDP-inducing activity in porcine IPEC-J2 cells and 3D4/31 macrophages.

**Table 2:** The Z-scores, fold increases of *pBD3* expression level in parental IPEC-J2 cells of 21 hits at indicated concentrations from primary and secondary screening of the natural product library

Compounds	Z-score, 20 μmol/L	Fold change			CAS number	Structural family	Source
		5 μmol/L	20 μmol/L	80 μmol/L			
Deoxyshikonin	6.79	20.75	3.84		43043-74-9	Quinones	*Lithosperraum erythrorhizon*
Scutellarein	4.73	0.68	3.30	1.29	529-53-3	Flavonoids	*Scutellaria baicalensis*
Pinostilbene	4.57	1.06	1.23	4.16	42438-89-1	Phenols	Pinaceae
Baicalin	4.16	1.13	3.14	2.21	21967-41-9	Flavonoids	*Scutellaria baicalensis*
Pterostilbene	4.00	1.33	1.57	4.38	537-42-8	Phenols	*Cyanococcus*
Tectorigenin	3.97	1.78	1.50	4.73	548-77-6	Flavonoids	*Belamcanda chinensis* (L.) DC.
Sophoricoside	3.88	1.02	2.73	1.76	152-95-4	Flavonoids	*Styphnolobium japonicum*
Piceatannol 3′-O-glucoside	3.74	1.41	1.90	3.03	94356-26-0	Phenols	*Rheum*
Isorhapontigenin	3.69	1.88	3.30	12.62	32507-66-7	Phenols	*Gnetum cleistostachynm*
6-Hydroxyflavone	3.55	1.36	2.91	5.90	6665-83-4	Flavonoids	Animal
Nocodazole	3.50	2.49	3.48	6.28	31430-18-9	Alkaloids	Plant
Irigenin	3.16	1.34	2.12	4.15	548-76-5	Flavonoids	*Belamcanda chinensis* (L.) DC.
Calycosin-7-O-beta-D-glucoside	2.90	1.32	1.23	1.28	20633-67-4	Flavonoids	*Astragalus membranaceus*
Dihydroresveratrol	2.84	0.79	1.66	1.13	58436-28-5	Phenols	*Veratrum nigrum*
Obtusifolin	2.74	1.33	1.77	4.86	477-85-0	Quinones	*Cassia angustifolia*
Nepetin-7-glucoside	2.61	0.96	1.11	1.76	569-90-4	Flavonoids	*Salvia plebeia R.Br.*
Calycosin	2.53	1.26	3.64	10.31	20575-57-9	Flavonoids	*Astragalus membranaceus*
Tectoridin	2.44	1.39	1.67	1.26	611-40-5	Flavonoids	*Belamcanda chinensis* (L.) DC.
Rhapontigenin	2.09	0.99	0.99	3.91	500-65-2	Phenols	*Rheum*
Xanthohumol	2.01	1.75	6.65	16.26	6754-58-1	Flavonoids	Plant
Polydatin	2.01	0.77	1.64	2.43	27208-80-6	Phenols	*Fallopia japonica*

### Validation of HDP-inducing compounds in porcine cell lines

**Fig. 2:**
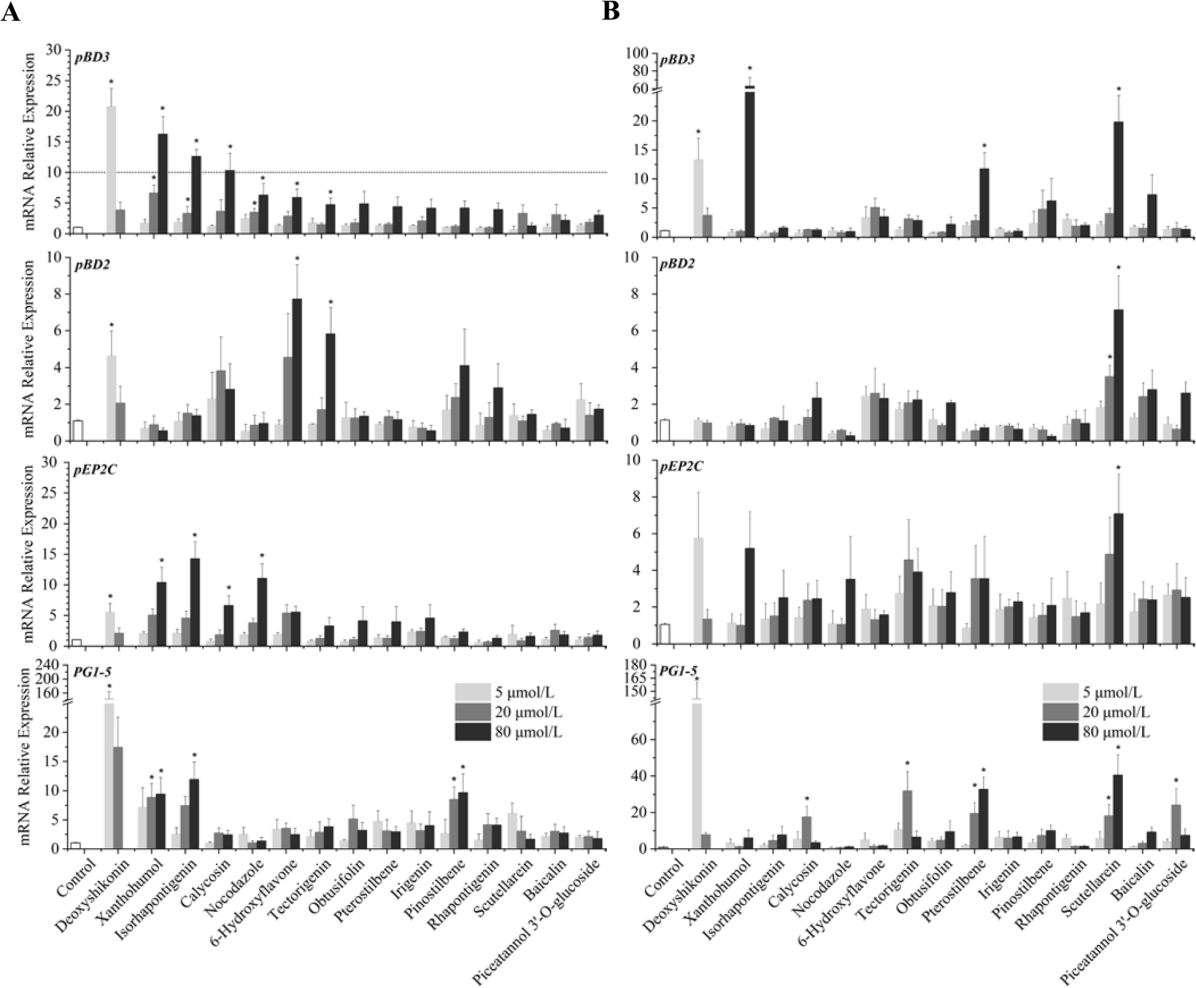
Confirmation of the natural products that induce mRNA expression of multiple porcine HDP genes in IPEC-J2 (**a**) and 3D4/31 cells (**b**). Cells were treated with or without different concentrations (5, 20, or 80 μmol/L) of the hits for 24 h, followed by RT-qPCR analysis of four representative porcine HDP genes (*pBD3*, *pBD2*, *PG1*-5, and *pEP2C*). The results are the mean ± SEM of three independent experiments. *P < 0.05 vs. non-treated control by unpaired Student’s *t*-test

To confirm the ability of the 21 hits to induce *pBD3* expression in the porcine cells, parental IPEC-J2 cells were firstly treated with each of 21 compounds at 5, 20, and 80 μmol/L for 24 h. As expected, most compounds were capable of inducing *pBD3* mRNA expression, albeit to different levels. Among them, 15 compounds gave a minimum of 3-fold increase in *pBD3* expression (Fig. 2a). Xanthohumol, isorhapontigenin, deoxyshikonin, and calycosin were the most potent in *pBD3* induction, showing a fold increase of > 10. To examine whether these compounds could also regulate other porcine HDP genes, we analyzed *pBD2*, *pEP2C*, and *PG1-5* expression in IPEC-J2 cells following stimulation. A majority of the compounds induced the expression of these three genes, albeit with varying efficacies. Interestingly, HDP genes exhibited a differential response to different compounds. For example, *pBD2* showed a generally reduced response, relative to *pEP2C* and *PG1-5*. Although deoxyshikonin was among the most potent in inducing all three porcine HDP genes, xanthohumol was highly effective in the induction of *pBD3*, *pEP2C* and *PG1-5*, with a negligible activity in *pBD2* induction. Of note, 5 μmol/L deoxyshikonin strongly induced *PG1-5* expression with a fold change of > 150, when compared with other 14 compounds that gave a maximum induction of 12-fold at any concentration examined.

To evaluate whether other porcine cell types are regulated by the 15 compounds, porcine 3D4/31 alveolar macrophages were stimulated with different concentrations of these compounds for 24 h, followed by RT-qPCR analysis of several HDP genes. Most compounds with the ability to induce HDP expression in IPEC-J2 cells enhanced the expression levels of multiple HDP genes in 3D4/31 macrophages. Deoxyshikonin and xanthohumol were again highly effective in inducing the expression of multiple HDP genes in 3D4/31 cells, similar to IPEC-J2 cells; however, several other potent compounds in IPEC-J2 cells lost their HDP-inducing activity substantially in 3D4/31 cells (Fig. 2b). On the other hand, scutellarein, a weak compound in IPEC-J2 cells, became highly efficacious in inducing all four HDP gene expression in 3D4/31 cells. These results clearly suggested a cell-specific regulation of HDP genes.

**Fig. 3:**
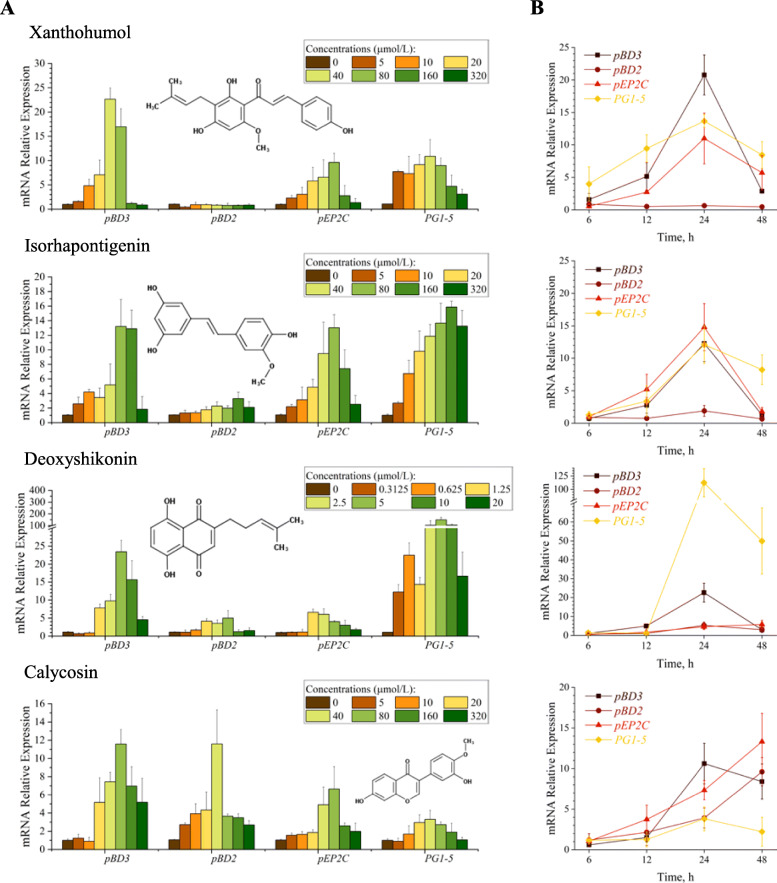
Induction of multiple porcine HDP expression in IPEC-J2 cells by xanthohumol, isorhapontigenin, deoxyshikonin, and calycosin in a dose (**a**) and time (**b**)-dependent manner. Chemical structure of each compound was also shown in panel **a**. IPEC-J2 cells treated with xanthohumol, Isorhapontigenin, or calycosin at 5 to 320 μmol/L, or with deoxyshikonin at 0.3125 to 20 μmol/L for 24 h, followed by RT-qPCR analysis of porcine HDP genes (**a**). IPEC-J2 cells treated with 40 μmol/L xanthohumol, 80 μmol/L isorhapontigenin, 5 μmol/L deoxyshikonin, and 80 μmol/L calycosin for 6, 12, 24, 48 h, followed by RT-qPCR analysis of porcine HDP genes (**b**). The results are the mean ± SEM of three independent experiments. *P < 0.05 vs. non-treated control by unpaired Student’s *t*-test

To identify optimal concentrations of four selected compounds in HDP induction, IPEC-J2 cells were treated with a broader concentration range of xanthohumol, isorhapontigenin, deoxyshikonin, and calycosin for 24 h, followed by RT-qPCR analysis of multiple HDP genes. The results indicated that 40 μmol/L xanthohumol was the best concentration to induce *pBD3* and *pG1-5*, whereas 80 μmol/L was the most effective in *pEP2C* induction (Fig. 3a). On the other hand, 80 μmol/L isorhapontigenin and calycosin as well as 5 μmol/L deoxyshikonin gave a peak induction of *pBD3*. Isorhapontigenin, deoxyshikonin and calycosin showed a maximum *pBD2* induction at 160, 5, 40 μmol/L, respectively, while xanthohumol failed to induce *pBD2* mRNA expression in IPEC-J2 cells. For *pEP2C*, 80 μmol/L xanthohumol, isorhapontigenin and calycosin led to a maximum induction, which occurred with 1.25 μmol/L deoxyshikonin. Notably, 5 μmol/L deoxyshikonin gave peak *PG1-5* expression with an over 150-fold increase. However, 40 μmol/L xanthohumol, 40 μmol/L calycosin, and 160 μmol/L isorhapontigenin exhibited the highest induction in *PG1-5* expression. It is noteworthy that higher concentrations of these compounds showed diminished HDP induction, suggesting the existence of a negative feedback mechanism. In follow-up time-course experiments, xanthohumol, isorhapontigenin, deoxyshikonin, and calycosin gave peak induction of most HDPs at 24 h, except that calycosin caused a maximum increase in *pBD2* and *pEP2C* expression at 48 h (Fig. 3b).

### Confirmation of HDP-inducing compounds in porcine Jejunal explants

**Fig. 4:**
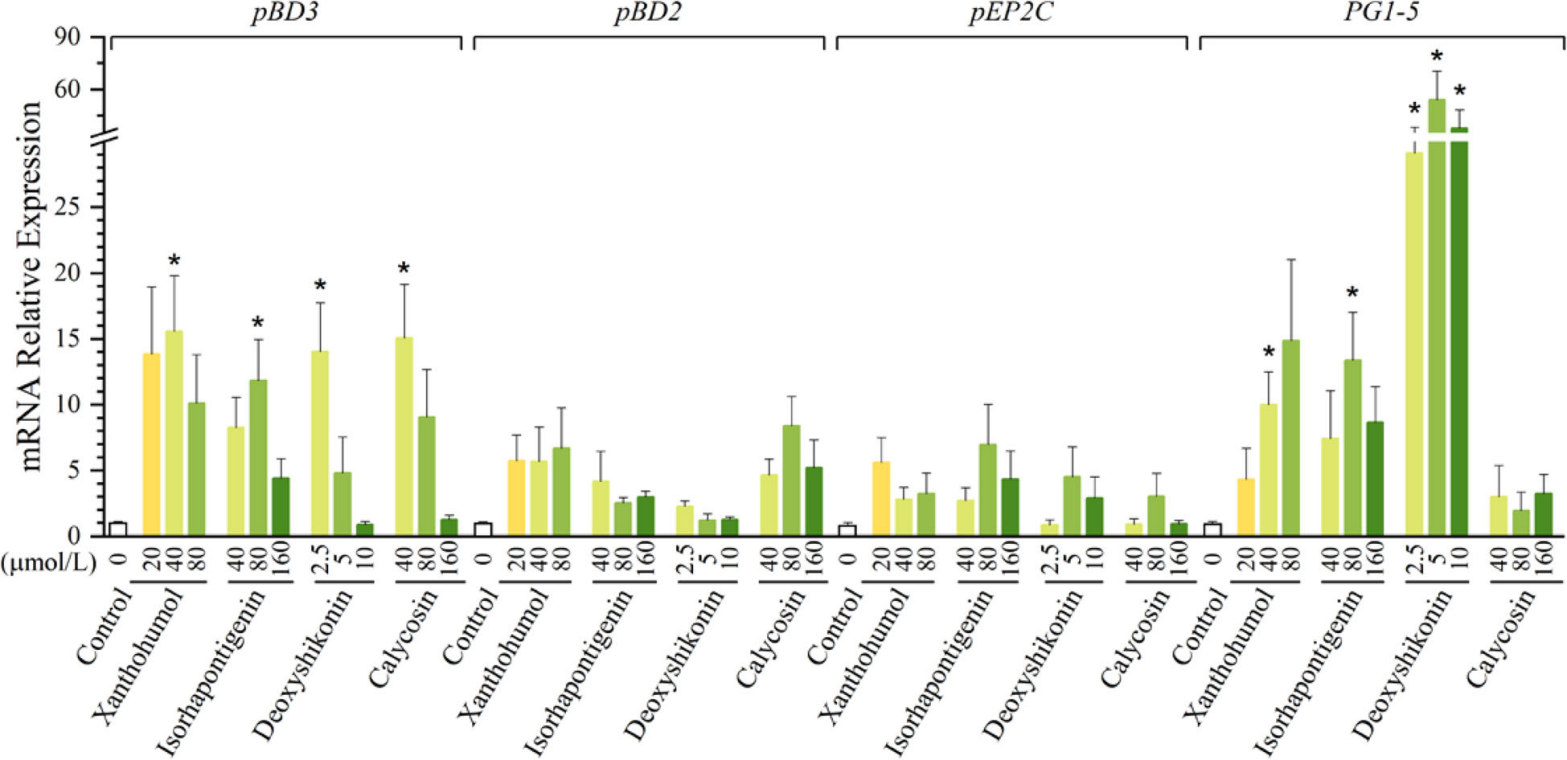
Induction of multiple porcine HDP expression in IPEC-J2 cells by xanthohumol, isorhapontigenin, deoxyshikonin, and calycosin in porcine intestinal explants. Porcine jejunal explants were treated with or without indicated concentrations of each compound for 24 h, followed by RT-qPCR analysis of *pBD3*, *pBD2*, *PG1-5*, and *pEP2C*. The results are the mean ± SEM of three independent experiments. *P < 0.05 vs. non-treated control by unpaired Student’s *t*-test

To confirm the ability of individual compounds to induce HDP expression in porcine primary intestinal cells *ex vivo*, jejunal explants were prepared from newly weaned pigs and stimulated with three different concentrations of xanthohumol, isorhapontigenin, deoxyshikonin, and calycosin for 24 h, followed by RNA isolation and RT-qPCR. As expected, all four compounds dose-dependently induced HDP gene expression in porcine jejunal explants (Fig. 4). Consistent with the results in IPEC-J2 and 3D4/31 cells, deoxyshikonin was the most potent in *PG1-5* induction in jejunal explants. Interestingly, xanthohumol, which was minimally effective in *pBD2* induction *in vitro*, was obviously effective *ex vivo* in jejunal explants. Overall, the HDP -inducing activity of four selected compounds were confirmed in porcine intestinal explants.

### Induction of Proinflammatory cytokine expression by selected compounds

**Fig. 5:**
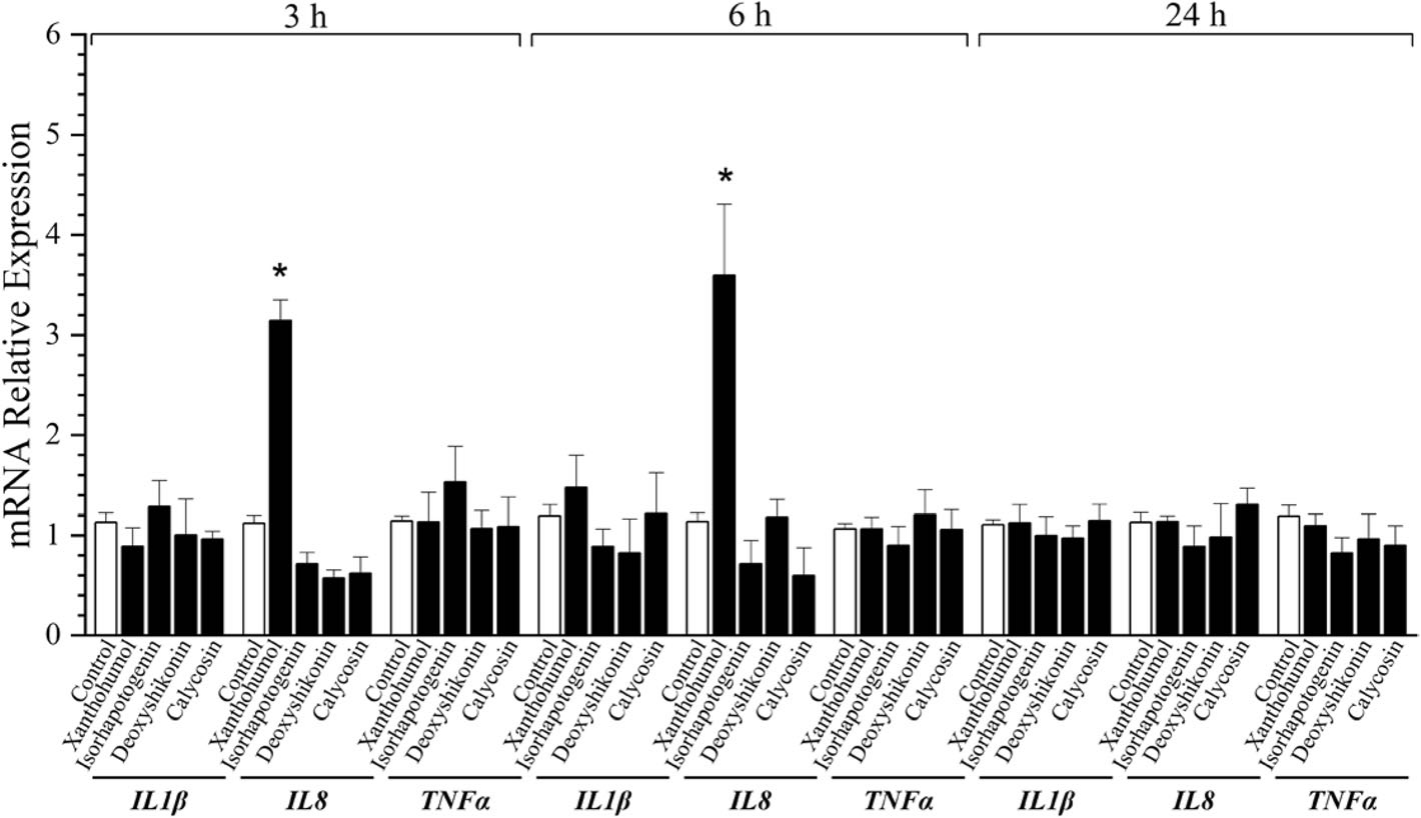
Minimum induction of proinflammatory cytokine expression by selected compounds. IPEC-J2 cells were treated with 40 μmol/L xanthohumol, 80 μmol/L isorhapontigenin, 5 μmol/L deoxyshikonin, or 80 μmol/L calycosin for 3, 6, and 24 h, followed by RT-qPCR analysis of *IL-1β*, *IL-8*, and *TNF-α* expression. The results are the mean ± SEM of three independent experiments. *P < 0.05 vs. non-treated control by unpaired two-tailed Student’s *t*-test

To examine whether newly-identified HDP-inducing natural products trigger inflammatory response, IPEC-J2 cells were stimulated with the optimal HDP-inducing dose of each compound for 3 h, 6 h, and 24 h, followed by RT-qPCR analysis of *IL1β*, *IL8*, and *TNFα* mRNA expression. As shown in Fig. 5, none of the proinflammatory cytokine expression was affected by any of the compounds (*P* > 0.05), except that xanthohumol triggered a mild *IL-8* expression only at 3 h and 6 h, suggesting that these natural products are capable of inducing HDP gene expression without provoking obvious inflammation.

### Enhancement of the antibacterial activity of porcine 3D4/31 cells by selected compounds

**Fig. 6:**
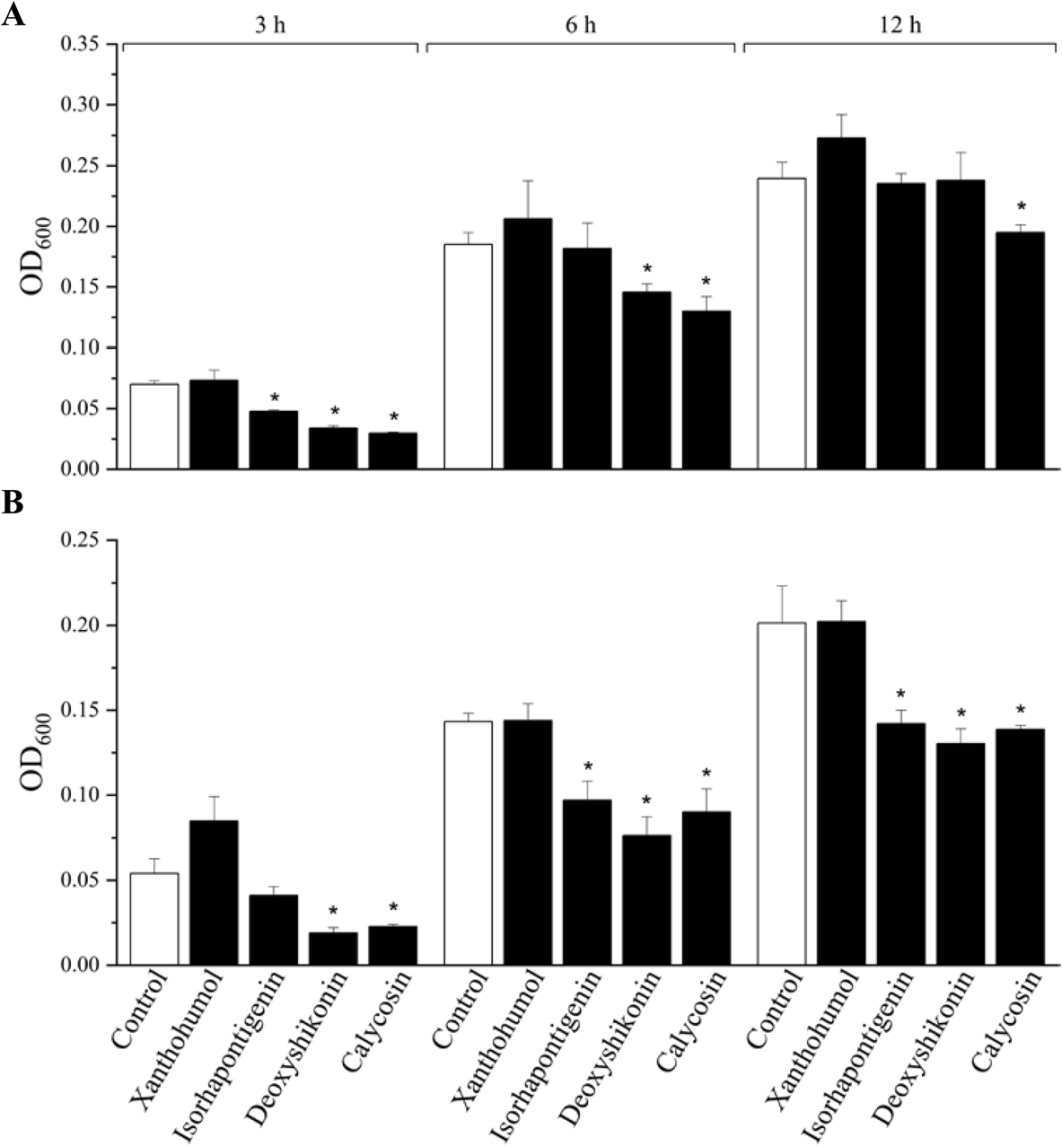
Augmentation of the antibacterial activity of porcine 3D4/31 macrophages by selected HDP-inducing compounds. Porcine 3D4/31 cells were stimulated with 40 μmol/L xanthohumol, 80 μmol/L Isorhapontigenin, 5 μmol/L deoxyshikonin, or 80 μmol/L calycosin for 12 h. Cell lysates were then incubated with ETEC or *S. aureus* at 37 °C. At 3, 6, and 12 h, the optical density at 600 nm was measured. The results are the mean ± SEM of three independent experiments. *P < 0.05 vs. non-treated control, evaluated by unpaired two-tailed Student’s *t*-test

To further evaluate natural product-induced HDP expression could lead to augmentation of the antibacterial activity of host cells, porcine 3D4/31 cells were stimulated with 40 μmol/L xanthohumol, 80 μmol/L isorhapontigenin, 5 μmol/L deoxyshikonin, or 80 μmol/L calycosin for 24 h followed by cell lysis. Bacterial turbidity was measured at 3, 6, and 12 h after incubation of the cell lysate with Gram-negative bacteria (ETEC) or Gram-positive bacteria (*S. aureus*). Isorhapontigenin, deoxyshikonin, and calycosin significantly suppressed the growth of both ETEC and *S. aureus*, while 40 μmol/L xanthohumol failed to inhibit bacterial growth in 3D4/31 cells at any time point (Fig. 6). In order to exclude the possibility that the enhanced antibacterial activity of porcine 3D4/31 cells was caused by the compounds themselves, their direct antibacterial activity (MIC) was determined using a standard broth microdilution assay. None of the four compounds showed an obvious antibacterial activity up to 320 μmol/L, implying that enhanced antibacterial activity of 3D4/31 cells was likely due to increased HDP synthesis but not their direct antibacterial activity.

## Discussion

The need for novel antibiotic alternatives that are less likely to trigger bacterial resistance has instigated an interest in the use of natural antimicrobials as they have a myriad of health-promoting properties. In this study, a screening of 1261 natural products has led to the identification of 48 compounds with a Z-score of > 2. Fifteen have been further confirmed to be potent in inducing *pBD3* mRNA expression. Of these, seven are flavonoids, five are phenols, two are quinones, and one is an alkaloid (Table 2). All but pterostilbene [22] have been reported to be able to induce HDP gene expression. Pterostilbene is a natural methoxylated analog of resveratrol [23] and both have been shown to induce human HDP gene expression [22, 24]. Perhaps not to our surprise, pinostilbene, a methylated derivative of resveratrol, and isorhapontigenin and rhapontigenin, two isomeric analogs of resveratrol, have also been identified to induce HDP gene expression in this study (Table 2). Pterostilbene and baicalin are epigenetic modulators by suppressing histone deacetylase (HDAC) 1 expression [25, 26], which contributes to histone hyperacetylation, and thus enhance gene expression [27]. Pterostilbene and scutellarein are also involved in DNA damage response [28, 29], which is known to play a positive role in gene transcription initiation [30, 31]. These compounds might regulate porcine HDP expression through epigenetic modification of HDP gene promoters.

Xanthohumol, isorhapontigenin, deoxyshikonin, and calycosin have been further validated to be among the most potent *pBD3*-inducing compounds in both IPEC-J2 and 3D4/31 cells as well jejunal explants. These four natural products belong to three different families, namely flavonoids, phenols, and quinones (Table 2). Xanthohumol, a prenylated chalcone naturally occuring in hops (*Humulus lupulus* L.), has attracted substantial attention owing to its abundant pharmacological activities including antimicrobial [32], anti-inflammatory [33], antioxidant [34], and anticancer activities [35]. This is the first study to reveal the HDP-inducing activity of xanthohumol. However, the mechanism by which xanthohumol induces HDP expression remains unclear. It is known that xanthohumol exerts chemo-preventive effects through inhibiting cyclooxygenase 1 and 2 activities [36]. Xanthohumol was also recently found to bind to histone H2A [37]. It will be interesting to study the involvement of cyclooxygenase inhibition and histone modifications in xanthohumol-mediated HDP induction.

Isorhapontigenin is a natural derivative of stilbene, which is present in numerous plant species. It has prominent anti-inflammatory, anti-cancer, and anti-diabetic potential [38–40]. As a resveratrol analog, isorhapontigenin is a tetrahydroxylated stilbenoid with a methoxy group. Given that resveratrol is a potent sirtuin 1 (SIRT1) activator, isorhapontigenin may also activate SIRT1. Indeed, isorhapontigenin shows a strong affinity for SIRT1 based on molecular docking and is more potent than resveratrol in SIRT1 activation [40], suggesting that isorhapontigenin might augment porcine HDP gene expression through activating SIRT1.

Deoxyshikonin is found in *Lithospermum erythrorhizon* and a promising drug candidate for the treatment of wounds and cancers [41, 42]. Deoxyshikonin is a derivative of shikonin, which is a well-known traditional Chinese medicine that has long been used for the treatment of burns, external wounds, infected crusts, and hemorrhoids owing to its numerous pharmacological properties [43, 44]. Shikonin is known to increase histone H3 acetylation [45] and induce FOXO3 phosphorylation [46]. How deoxyshikonin transcriptionally activates porcine HDP genes expression remains to be investigated.

Calycosin, an isoflavonoid, is found in *Astragalus membranaceus*, a traditional Chinese medicinal herb. Calycosin has anti-oxidative, anti-inflammatory, and anti-tumorigenic properties [47–49]. Similar to isorhapontigenin, calycosin is also a potent activator of SIRT1 [50], suggesting that calycosin may induce porcine HDP expression through SIRT1 activation.

In our study, the compounds were initially identified based on their ability to enhance *pBD3* gene expression. Desirably, they all have the capacity to induce multiple other HDP genes such as *pBD2*, *pEP2C*, and *PG1-5*, albeit with varying efficacies, which may be at least in part explained by a difference in the promoter structure of individual HDP genes. Obviously, HDP genes are also regulated in a cell-specific manner, and a single HDP gene is differentially modulated by the same compound in different cell types. This result is consistent with findings for other compounds in our previous studies [14]. Similar observations have also been reported for human cathelicidin *LL-37*, which is strongly induced by butyrate in intestinal epithelial cells, but minimally regulated in monocytes or skin keratinocytes [51].

Due to a lack of commercial antibody to any of the porcine HDPs, we could not directly verify increased HDP synthesis at the protein level following compound stimulation of porcine cells. Instead we assayed for a change in the antibacterial activity of host cells. Consistent with our expectation, three out of four compounds significantly suppressed the growth of both Gram-positive and Gram-negative bacteria in porcine macrophages following 24-h treatment. Our results are also in agreement with the ability of other HDP-inducing compounds to augment the antibacterial activity of host cells [16, 21, 52]. However, it is noted that an increase in the antimicrobial potency of host cells may not be entirely due to the HDP-inducing activity of the compounds. Nevertheless, these compounds, when used at HDP-inducing concentrations, show no obvious direct antibacterial activity, suggesting that they have potential to enhance HDP synthesis and animal immunity with a minimum risk of triggering bacterial resistance.

## Conclusions

In conclusion, we have successfully identified several natural products that can induce porcine HDP expression following a HTS assay. The HDP-inducing effect of these compounds have been thoroughly demonstrated in two different porcine cell lines and jejunal explants. With their ability to potentiate the antibacterial activity of host cells, these HDP-inducing natural products show the potential to be developed as novel alternatives to antibiotics for disease control and prevention in pigs and possibly other livestock.

## Data Availability

The data generated and/or analyzed during the current study are available from the corresponding authors upon request.

## Abbreviations

ETEC: Enterotoxigenic *E. coli*
GAPDH: Glyceraldehyde-3-phosphate dehydrogenase
HDAC: Histone deacetylase
HDP: Host defense peptides
HTS: High throughput screening
MIC: Minimum inhibitory concentration
NCCLS: National Committee for Clinical Laboratory Standards
SEM: Standard errors of the mean
SIRT1: Sirtuin 1
